# Synergistic Oncolytic Effect of HSVtk- and IL-15Rα-Armed Vaccinia Viruses Inducing Systemic Antitumor Immunity

**DOI:** 10.3390/ijms27135838

**Published:** 2026-06-28

**Authors:** Olga N. Alekseeva, Pavel O. Vorobyev, Yasmin Shakiba, Stepan A. Ionov, Svetlana S. Antseva, Anastasia V. Semenova, Marat P. Valikhov, Vladimir A. Kalsin, Veronika V. Vadekhina, Dmitry V. Kochetkov, Peter M. Chumakov, Anastasia V. Poteryakhina

**Affiliations:** 1 Engelhard Institute of Molecular Biology, Russian Academy of Science, 119991 Moscow, Russia; yasi.shakiba@gmail.com (Y.S.); stephan.ionov@yandex.ru (S.A.I.); sveta.antseva@yandex.ru (S.S.A.); n.semenowa2016@yandex.ru (A.V.S.); marat.valikhov@gmail.com (M.P.V.); dvkochetkov@gmail.com (D.V.K.); chumakovpm@yahoo.com (P.M.C.); lipatovaanv@gmail.com (A.V.P.); 2 Moscow Center for Advanced Studies, Kulakova Str. 20, 123592 Moscow, Russia; 3 Federal Scientific and Clinical Center for Specialized Types of Medical Care and Medical Technologies, Federal Medical and Biological Agency, 115682 Moscow, Russia; vkalsin@mail.ru; 4 Serbsky National Medical Research Center for Psychiatry and Narcology, Ministry of Health of the Russian Federation, 119034 Moscow, Russia; veronikavadehina@gmail.com; 5 Department of Medical Nanobiotechnology, Pirogov Russian National Research Medical University, Ostrovitianov Street, 1, 117997 Moscow, Russia

**Keywords:** oncolytic virus, vaccinia virus, thymidine kinase, interleukin 15

## Abstract

Oncolytic virotherapy offers a promising avenue for solid tumor treatment, yet single-agent approaches are frequently limited by insufficient tumor lysis and inadequate immune activation. Here we report that combined therapy with two recombinant variants of the oncolytic vaccinia virus, armed with either herpes simplex virus thymidine kinase (VV-HSVtk) or the interleukin 15 receptor subunit alpha (VV-mIL15Rα), leads to enhanced cytotoxicity and immune stimulation in a murine mammary adenocarcinoma model (4T1). In vitro, VV-HSVtk exhibited dose-dependent cytotoxicity markedly potentiated by ganciclovir (GCV) through HSVtk-mediated phosphorylation into a cytotoxic nucleoside analog, and co-culture of VV-infected tumor cells with donor-derived NK cells further increased oncolytic efficiency. In vivo, combined treatment with VV-HSVtk, VV-mIL15Rα, and GCV resulted in significant tumor regression and extended survival relative to monotherapy controls in 4T1 syngeneic mice. Histological examination revealed increased lymphocytic infiltration at tumor sites and absence of hepatic or splenic toxicity. Together, these data indicate that integrating direct viral cytotoxicity, HSVtk/GCV-mediated suicide gene therapy, and IL-15-pathway-targeted immunomodulation within an oncolytic vaccinia platform can improve antitumor efficacy in a stringent breast cancer model.

## 1. Introduction

Oncolytic virus (OV) therapy has emerged as a transformative modality in precision oncology, utilizing viruses that selectively replicate within and lyse malignant cells while sparing healthy tissues [1,2]. Beyond direct oncolysis, OVs function as potent immunostimulatory agents capable of reversing the immunosuppressive tumor microenvironment (TME) and priming systemic anti-cancer immunity [3]. This therapeutic potential is exemplified by T-VEC, a GM-CSF-encoding herpes simplex virus and the first FDA-approved OV for melanoma [4]. Among the various viral platforms, vaccinia viruses (VVs) are particularly advantageous due to their significant transgene-encoding capacity, robust safety profile, and natural tropism for neoplastic tissues [5].

A primary approach to enhance VV potency involves integrating immunostimulatory transgenes, like cytokines, to connect direct oncolysis with broad systemic antitumor immunity [6,7]. Interleukin-15 (IL-15) is a pleiotropic cytokine essential for the development, activation, and survival of cytotoxic T cells and natural killer (NK) cells [8]. Its biological activity is dramatically enhanced when complexed with the interleukin-15 receptor subunit alpha (IL-15Rα), which mediates high-affinity signaling and promotes long-term lymphoid survival [9]. Recent studies have demonstrated that VVs encoding IL-15 or IL-15/IL-15Rα fusion proteins can induce remission in various cancer models, particularly when combined with checkpoint inhibitors [10,11].

In addition to immunotherapy, metabolic “suicide gene” therapy (such as herpes simplex virus type 1 thymidine kinase—HSVtk system) offers a powerful method to enhance local tumor destruction [12,13]. When combined with the prodrug ganciclovir (GCV), HSVtk converts the drug into a cytotoxic triphosphate form that inhibits DNA synthesis [14]. This system is characterized by a “bystander effect,” in which the toxic metabolites spread to neighboring HSVtk-negative (non-expressing) tumor cells, significantly amplifying the viral oncolytic footprint [15].

In this study, we utilized the Lister strain of vaccinia virus from the Institute of Viral Preparation (LIVP), an attenuated variant with established efficacy for treatment of highly immunosuppressive models like triple-negative breast cancer (TNBC) [16,17]. We engineered two recombinant variants: VV-HSVtk and VV-mIL15Rα, with a disrupted VV thymidine kinase gene and RFP expression within a bicistronic cassette alongside the target transgene [11].

In the present study, we investigated whether the combined use of a metabolically armed HSVtk-expressing LIVP variant and an immunostimulatory mIL15Rα-expressing LIVP variant, together with GCV, could improve local tumor destruction and enhance systemic antitumor immune responses. The therapeutic efficacy of this multimodal approach was assessed in vitro across murine and human tumor cell models and in vivo in the syngeneic 4T1 breast carcinoma model.

## 2. Results

### 2.1. Construction and Characterization of Recombinant LIVP Variants

**Figure 1:**
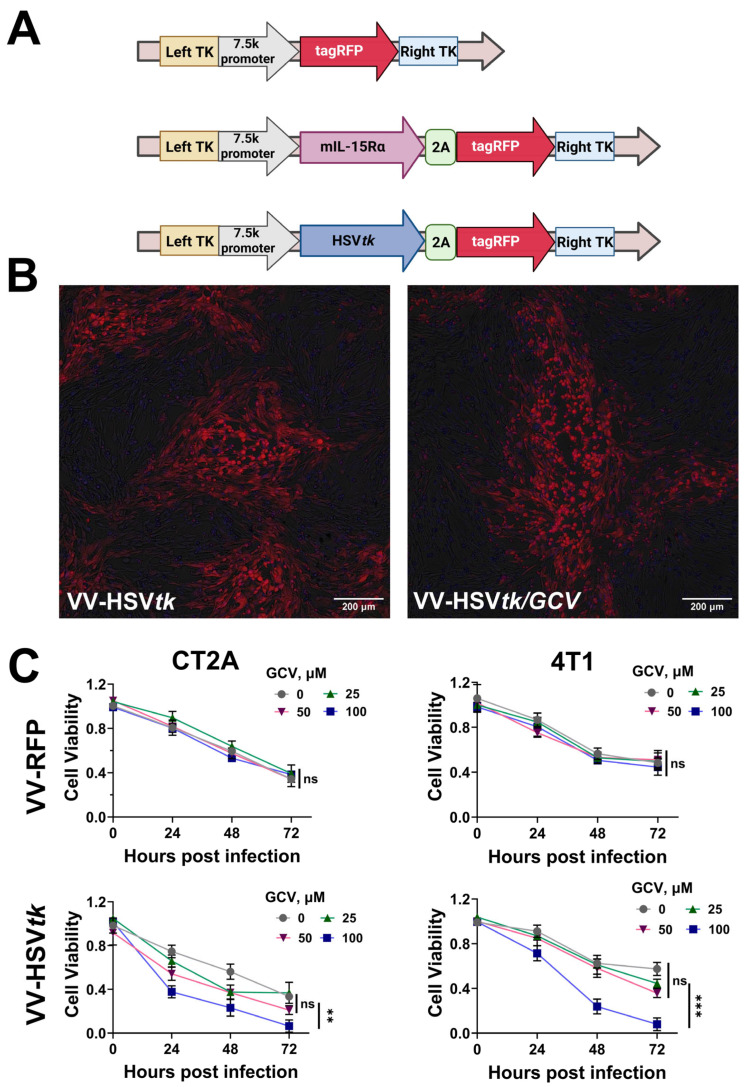
(**A**) Scheme of genetic modification of the TK gene locus; (**B**) microphotographs of viral plaques on BHK-21 cells; (**C**) comparison of cytotoxic effect of VV-RFP and VV-HSVtk strains in the presence of GCV in the range of concentrations on CT2A and 4T1 cell lines at MOI = 10 (n = 3 independent biological replicates per condition). Data are presented as mean ± SD. Statistical analysis was performed using the Mann–Whitney U-test; ns—not significant, ** *p* < 0.01, *** *p* < 0.001.

The recombinant vaccinia virus variants were engineered via homologous recombination, targeting the viral thymidine kinase (TK) locus to enhance tumor selectivity [5]. The reporter gene tagRFP was utilized for visualization of viral replication and efficacy of transgene expression (Figure 1A). Successful integration of expression cassettes into the LIVP genome was confirmed by Sanger sequencing of the amplified TK locus.

Infectivity and cytolytic effect of the variants were dynamically assessed on BHK-21, CT2A, and 4T1 cells (Supplementary Figure S1). Fluorescence microscopy confirmed that both VV-HSVtk and VV-mIL15Rα effectively infected and replicated within these cell lines (Figure 1B,C). RT-PCR confirmed robust expression of HSV1 thymidine kinase in lysates from VV-HSVtk-RFP-infected cells, laying the groundwork for GCV-induced cytotoxicity (Supplementary Figure S2). Kinetic properties of the VV-mIL15Ra variant were described in detail earlier [11].

### 2.2. LIVP Recombinant Strains Demonstrate Enhanced Oncolysis via Metabolic Cooperation and NK Cell Recruitment

Treating immunologically “cold” tumors remains a formidable challenge in oncology due to a hostile TME characterized by poor infiltration of effector cells and a lack of inflammatory signaling. To address this, we engineered an oncolytic platform designed to “heat up” these non-immunogenic environments through a multimodal attack [18]. We performed an assessment of its efficacy across a diverse panel of human cancer cell lines, tracking cytotoxicity at 24, 48, 72, and 96 h post-infection. The integration of the VV-HSVtk strain, when combined with GCV, catalyzed a profound dose-dependent escalation in cytotoxicity that effectively surpassed the baseline oncolytic activity of the VV-RFP variant. As observed at GCV concentration of 100 μM, cell viability was drastically compromised, validating the successful activation of a potent bystander effect. This allows toxic molecules to diffuse through gap junctions to eliminate adjacent uninfected cells, effectively circumventing the barriers to viral spread often found in dense immunosuppressive tumors [19].

**Figure 2:**
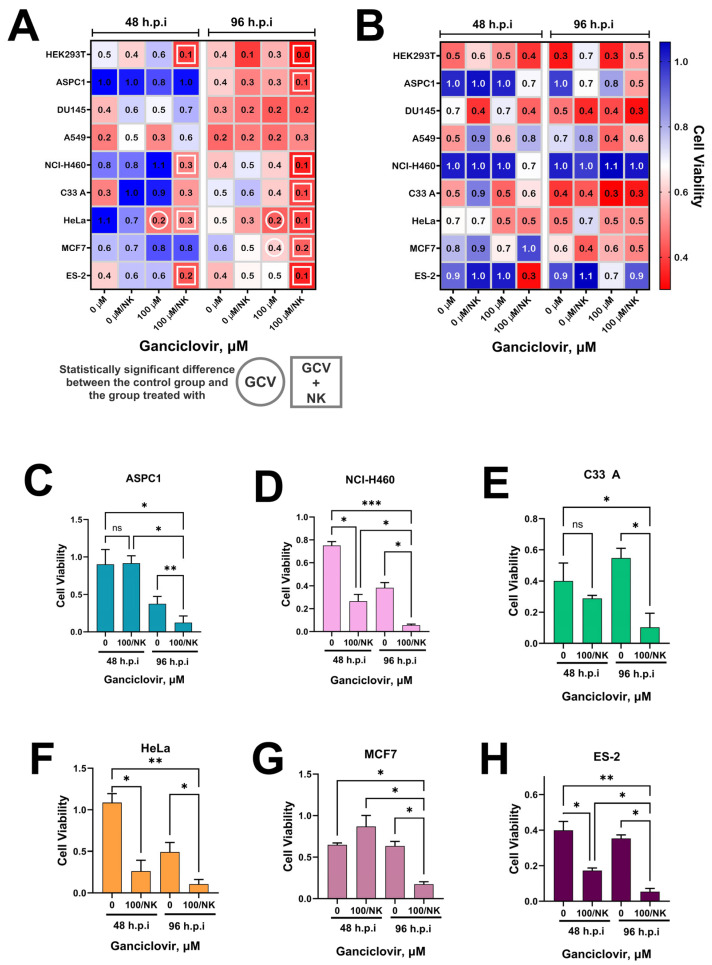
(**A**) Heatmap of cell viability after infection with VV-HSVtk at 48 and 96 h post-infection (h.p.i.). Combined treatment included the addition of GCV in a concentration of 100 μM and donor-derived human NK cells (n = 3 independent biological replicates per condition). Circles indicate statistically significant differences between replicates with and without GCV, and squares indicate the presence or absence of NK cells. (**B**) Heatmap of cell viability after infection with control strain VV-RFP (n = 3 independent biological replicates per condition). (**C**–**H**) Comprehensive comparison of cell viability in different model cell lines treated with or without adding a combination of GCV and NK cells after 48 and 96 h.p.i. (n = 3 independent biological replicates per condition). Data are presented as mean ± SD. Statistical analysis was performed using the two-way ANOVA; * *p* < 0.05, ** *p* < 0.01, and *** *p* < 0.001.

Beyond direct metabolic lysis, our aim is to reprogram the TME to favor innate immune engagement. By co-culturing human cancer lines—including HeLa, HEK293T, HCT116, NCI-H460, TOV21G, DU145, MCF7, and C33A—with primary human NK cells, we observed that the presence of these effector cells significantly accelerated the destruction of tumor cells infected with VV-HSVtk (Figure 2A,B). The presence of NK cells significantly enhanced the elimination of tumor cells compared with virus treatment alone, whereas this effect was less pronounced in control samples infected with VV-RFP. These findings indicate that metabolic cytotoxicity induced by HSVtk/GCV can be further complemented by immune effector cell activity. This transformation of the “cold” tumor landscape into a pro-inflammatory site facilitates the recruitment and activation of NK cells, providing a robust strategy for overcoming the inherent resistance of immunosuppressive malignancies [20]. Importantly, these co-culture experiments were designed as a mechanistic in vitro system to test whether our oncolytic platform creates a tumor context more permissive to NK-mediated killing, rather than as a direct surrogate for the murine NK compartment in BALB/c 4T1 tumors.

### 2.3. Combined Therapy with VV-HSVtk, VV-mIL15Rα, and Ganciclovir Induces Synergistic Tumor Regression In Vivo

**Figure 3:**
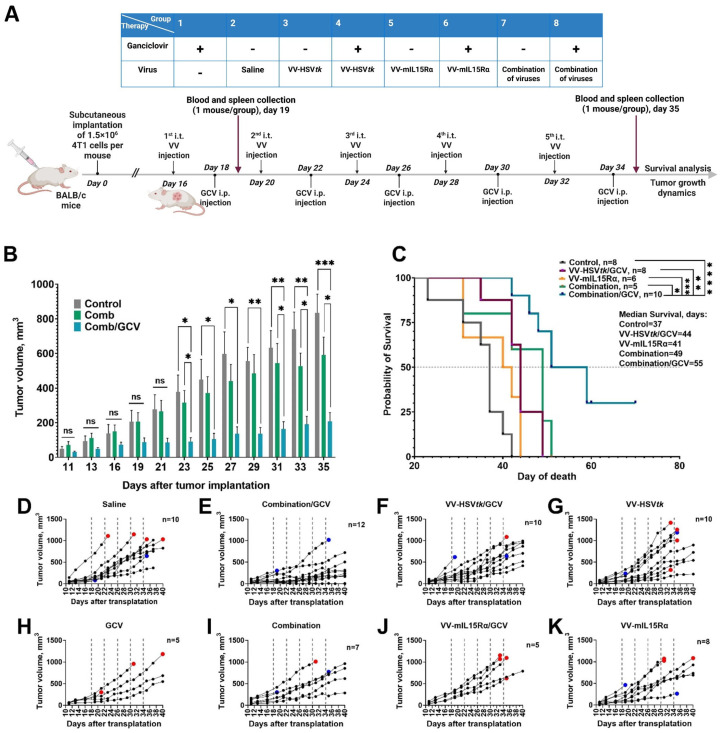
(**A**) Scheme of in vivo experiment; (**B**) dynamic of tumor growth; (**C**) Kaplan–Meier survival curves; (**D**–**K**) tumor volume growth curves for individual mice in each treatment group: (**D**)—control group, (**E**)—VV-HSVtk + VV-mIL15Ra + GCV, (**F**)—VV-HSVtk + GCV, (**G**)—VV-HSVtk, (**H**)—GCV, (**I**)—VV-HSVtk + VV-mIL15Ra, (**J**)—VV-mIL15Ra + GCV, and (**K**)—VV-mIL15Ra. Blue circles indicate animals that were electively sacrificed for tissue collection, whereas red circles indicate animals that reached the tumor volume endpoint (tumor volume ≥ 1000 mm^3^) or died spontaneously. Data are presented as mean ± SD. Statistical analysis of tumor growth was performed using two-way ANOVA, and survival was analyzed using the log-rank test; * *p* < 0.05, ** *p* < 0.01, *** *p* < 0.001, and **** *p* < 0.0001.

To evaluate the therapeutic potential of the recombinant strains, syngeneic mice bearing 4T1 breast carcinoma were treated with virotherapeutic agents alone or in combination once tumors reached a volume of approximately 50 mm^3^. The treatment regimen consisted of five intratumoral injections administered every four days. In groups receiving GCV, the prodrug was administered 48 h following each viral injection to allow for sufficient expression of the HSVtk transgene (Figure 3A). Tumor growth kinetics (Figure 3D–K) revealed that all viral treatment groups experienced reduced tumor progression compared to the Control and GCV-only control groups. While monotherapies with VV-HSVtk or VV-mIL15Rα showed moderate inhibitory effects, the addition of GCV to the VV-HSVtk group significantly enhanced local tumor debulking, confirming the in vivo efficacy of the metabolic suicide gene system. Notably, the VV-mIL15Rα/GCV group did not show significant improvement over VV-mIL15Rα alone, as expected given the absence of the prodrug converting transgene.

The strongest therapeutic effect was observed in the triple combination group receiving VV-HSVtk, VV-mIL-15Rα, and GCV, showing the slowest tumor progression (Figure 3B), the longest median survival among all treatment groups (Figure 3C), and even one complete tumor regression. In contrast, monotherapy and dual-treatment groups demonstrated moderate growth inhibition. These findings suggest that HSVtk/GCV-mediated metabolic cytotoxicity and mIL-15Rα-associated immune stimulation act cooperatively to improve tumor control in the highly aggressive 4T1 breast carcinoma model. Notably, 35% of mice in the combination therapy group demonstrated durable tumor growth arrest, with sustained suppression of tumor progression observed throughout the 70-day follow-up period; remarkably, one animal exhibited complete tumor regression, suggesting the induction of a curative antitumor immune response in a subset of treated subjects (Supplementary Figure S3). It is particularly notable, as 4T1 is widely regarded as one of the most immunosuppressive and treatment-resistant preclinical breast cancer models, characterized by profound CD8^+^ T cell exclusion and exhaustion [21]. To formally test for synergy in vivo, we analyzed the comb/GCV treatment using a Bliss independence framework applied to tumor volume measurements over time. The expected additive effect was derived from the tumor growth inhibition achieved by each monotherapy, and this Bliss-expected tumor volume trajectory was compared with the observed trajectory under comb/GCV treatment. The observed triple combination therapy tumor growth inhibition was significantly greater than the Bliss-expected additive effect (psyn = 0.0180), indicating a more-than-additive (synergistic) interaction between VV-HSVtk/GCV and VV-mIL15Rα in this model. Qualitative analysis for metastatic involvement of the liver (Supplementary Figure S4) indicates a modest reduction in the number of animals with metastatic lesions compared to the control groups, although the differences were not statistically significant. To assess intratumoral viral presence after treatment, we quantified vaccinia viral DNA in tumor samples collected 24 h and 72 h after injection of the recombinant VV vector. Viral genomes were readily detectable in tumors 24 h after treatment, and by 72 h, viral DNA copy numbers in treated tumors were more than 10 times lower than at 24 h, although viral genomes remained detectable in all tested samples, indicating that the VV establishes an early intratumoral presence that is partially lost over time (Supplementary Figure S5).

### 2.4. Systemic Immune Activation: Enhanced T Cell Function

FluoroSpot analysis of IFN-γ- and IL-2-secreting T cells in PBMC revealed a transient decrease in the number of antigen-specific T cells across all virus-treated groups following the first injection (Supplementary Figure S6). This early reduction is in line with the active redistribution of activated T cells from peripheral circulation into the tumor site and draining lymph nodes, rather than immune suppression, and is further supported by the concurrent systemic IFN-γ suppression observed in serum cytokine profiling at the same time point. The parallel decline in IL-2-secreting T cells suggests that peripheral T cells had undergone activation-driven consumption of IL-2 and subsequent trafficking to the TME, where local proliferation would not be captured by PBMC sampling. By the fifth injection, the triple combination therapy group demonstrated higher counts of both IFN-γ- and IL-2-secreting T cells compared to all other treatment groups. This late-phase superiority suggests that the combination regimen may have generated a more robust systemic pool of tumor-educated effector T cells, rather than definitively proving it, in a manner compatible with the combined effects of GCV-associated immunogenic cell death—which likely broadens tumor antigen availability for T-cell priming—and VV-mIL15Rα–mediated modulation of the IL-15 pathway, which could further support CD8^+^ memory T-cell and NK-cell survival. The magnitude of peripheral T-cell reconstitution in the combination group paralleled the most pronounced tumor volume reduction across all experimental arms, providing cellular-level observations that are compatible with, but do not by themselves prove, the superior therapeutic efficacy of this regimen.

### 2.5. Systemic Cytokine Response Following Repeated Administration of Recombinant LIVP Strains

**Figure 4:**
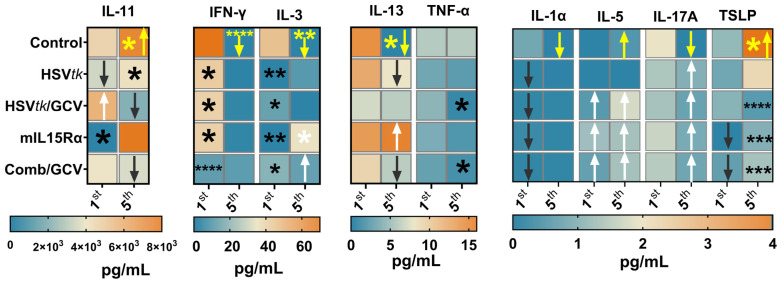
Assessment of cytokine dynamics in the serum of treated animals, grouped by average concentration from highest to lowest (left to right), with concentration scales shown below the heatmap. The color intensity of each cell in the heatmaps represents the mean cytokine concentration for the corresponding group and time point. Arrows denote changes greater than 30% that, due to the limited sample size, did not reach statistical significance but suggest biologically relevant trends. Yellow shading highlights changes observed in the control group between the first and fifth injections, whereas white arrows indicate increases and black arrows indicate decreases relative to the corresponding control group. Statistical analysis was performed using two-way ANOVA, and survival was analyzed using the log-rank test; * *p* < 0.05, ** *p* < 0.01, *** *p* < 0.001, and **** *p* < 0.0001.

To characterize the systemic immune landscape triggered by combined virotherapy, serum cytokine profiles were analyzed 24 h post-prime (1st injection) and post-boost (5th injection) in 4T1 tumor-bearing mice. The cohorts were treated with VV-HSVtk, VV-mIL15Rα, or their combinations, with or without GCV. The evaluated multiplex panel encompassed pro-inflammatory mediators, Th1-associated cytokines, Th17/epithelial alarmins, and immunoregulatory factors (Figure 4 and Supplementary Figure S7).

Overall, therapeutic intervention elicited a robust systemic cytokine flux; however, the profile diverged from a canonical cytotoxic antitumor signature. Instead, the response manifested as a complex hybrid state characterized by acute inflammatory activation, partial Th1/NK-cell stimulation, and the concomitant induction of immunoregulatory and tumor-supportive mediators.

Serum cytokine profiling revealed that treatment groups induced distinct and heterogeneous immune signatures rather than a uniform cytokine response. Following the first therapeutic injection, the VV-mIL15Rα group exhibited elevated IL-5 alongside reduced IFN-γ, IL-1α, IL-3, and TSLP; also, it was defined by lower levels of IL-4, IL-9, IL-11, and GM-CSF, but not so dramatically as others (Supplementary Figure S7). The VV-HSVtk group was characterized by decreased IFN-γ, IL-1α, and IL-3. The VV-HSVtk + GCV and triple combination groups additionally elevated IL-5 (however, without statistically significant differences with the control group).

At the end of therapy, when pronounced antitumor effects were observed, differences in serum cytokine concentrations relative to the control group were less obvious overall, yet qualitatively more informative. Following the fifth injection, the VV-mIL15Rα group exhibited elevated IL-3, IL-5, IL-13, and IL-17A, with reduced TSLP. The VV-HSVtk group was characterized by increased IL-22, alongside decreased IL-11 and TSLP. The VV-HSVtk/GCV group showed decreased TSLP and IL-11, together with elevated IL-5 and IL-17A. The combination of both viruses with GCV increased IL-3, IL-5, and IL-17A, while decreasing TSLP and IL-11. Taken together, these data demonstrate that the dual-virus combination with GCV uniquely integrates cytokine changes that are only partially recapitulated by each monotherapy arm, suggesting synergistic and mechanistically distinct immune remodeling that correlates with the observed dramatic reduction in tumor volume and complete responses in a subset of treated animals.

### 2.6. Combined Therapy Leads to Enhanced Tumor Necrosis and Immune Infiltration

**Figure 5:**
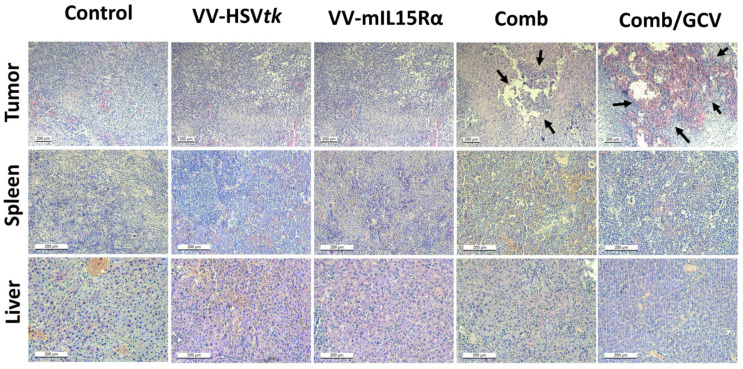
Histological analysis of tumor, spleen, and liver in treated animals; necrosis foci are marked with arrows.

The histological analysis of tumors (Figure 5, Supplementary Figure S8) in the combination and combination/GCV groups is consistent with enhanced local tumor destruction in these arms, with the absence of spleen and hepatic pathology. This result is aligned with the previously described safety profile of the LIVP strain, which shows natural selectivity for tumor cells [22]. In prior studies, HSVtk-based suicide gene therapy combined with cytokine gene strategies increased antitumor efficacy and could enhance both local and distant tumor control, supporting the biological plausibility of the current findings. Overall, these findings indicate that the combined treatment was associated with a heterogeneous cytokine response involving simultaneous changes in inflammatory, Th-associated, and immunoregulatory mediators, together with altered immune-cell infiltration in the TME [23].

Immunohistochemical staining for CD4 and CD8 (Supplementary Figures S9–S12) further supported cytokine profiling data by showing treatment-dependent changes in the tumor immune infiltrate. In tumors of control animals, a specific CD4 or CD8 signal was essentially absent, indicating a lack of appreciable T-cell infiltration. In contrast, the combination/GCV group showed a more pronounced presence of both CD4^+^ and CD8^+^ cells, suggesting stronger recruitment of adaptive immune cells into the tumor site. The HSVtk/GCV group also showed CD4^+^ and CD8^+^ cells, but the number of cells and the signal were weaker than in the combination/GCV group, indicating a more limited level of T-cell infiltration. This pattern was congruent with the increased IFN-γ response observed in the combination/GCV group and with the elevated IL-2 production detected in spleen cells at the end of treatment, supporting systemic T-cell activation. The pattern matches the mixed cytokine profile observed in serum, including increased IL-4, IL-5, and IL-17A together with a decrease in IFN-β and TSLP. Overall, these findings indicate that treatment altered both the systemic cytokine milieu and the local cellular composition of the TME.

## 3. Discussion

The present study demonstrates that integration of metabolic suicide gene therapy with cytokine-receptor modulation within the LIVP vaccinia virus backbone establishes a synergistic therapeutic window that neither strategy achieves independently. Although oncolytic viruses are inherently immunogenic [24,25], single-agent oncolytic virotherapy is frequently constrained by antiviral host defense mechanisms, acquired resistance, and intrinsic tumor heterogeneity, and the physical barriers imposed by extracellular matrix remodeling, stromal fibrosis, and tumor necrosis [26,27,28,29]. Our findings indicate that the triple combination of VV-HSVtk, VV-mIL15Rα, and GCV enhances antitumor efficacy by coupling direct tumor reduction with evidence of systemic immune activation, consistent with partial reversal of systemic immune evasion. To formally assess whether this interaction exceeds additivity, we applied a Bliss independence framework to in vivo tumor volume data and observed that the tumor growth inhibition achieved by the triple combination regimen significantly exceeded the Bliss-expected additive response (psyn = 0.0180). These results provide statistical evidence of a more-than-additive, synergistic interaction between VV-HSVtk/GCV and VV-mIL15Rα at the tested dosing schedule.

The primary obstacle in treating aggressive models such as 4T1 triple-negative breast cancer is the profoundly immunosuppressive TME, which actively excludes and exhausts infiltrating effector lymphocytes [30,31]. Our in vitro data establish the potency of the HSVtk/GCV bystander effect as the mechanistic foundation of this approach. In contrast to conventional oncolysis, which requires productive viral infection of individual tumor cells, HSVtk-mediated phosphorylation of GCV generates diffusible cytotoxic nucleoside analogs capable of destroying neighboring non-infected malignant cells [15,32]. This metabolic direct cell death not only reduces tumor burden but also releases a coordinated wave of tumor-associated antigens (TAAs) and damage-associated molecular patterns (DAMPs)—including HMGB1 and extracellular ATP, thereby supporting a shift from an immunologically “cold” to a more inflamed, antigen-rich environment that is permissive to adaptive immune priming. While we did not directly quantify DAMP release or dendritic cell activation, this interpretation is consistent with prior work on HSVtk/GCV and other immunogenic cell-death-inducing therapies [25,33,34,35,36].

However, antigen release alone is insufficient to sustain long-term remission when the host immune system remains functionally suppressed. This is the critical niche occupied by VV-mIL15Rα as a part of combined therapy. Unlike soluble IL-15, which is subject to rapid proteolytic degradation and limited bioavailability in the circulation, expression of the membrane-anchored IL-15 receptor α subunit facilitates the assembly of high-affinity IL-15/IL15Rα trans-presentation complexes on the surface of antigen-presenting cells, delivering a qualitatively superior and spatially focused activation signal to NK cells and CD8^+^ T cells [37,38,39].

A notable finding from our in vitro co-culture experiments was that, while viral infection alone did not fully account for the extent of tumor cell loss, the addition of donor-derived NK cells produced a marked increase in tumor cell death. This dissociation between viral cytolytic activity and immune-mediated killing supports a model in which oncolytic infection and metabolic debulking can render tumor cells more susceptible to NK-cell-mediated clearance. In the context of the full combination regimen in vivo, VV-mIL15Rα-mediated engagement of the IL-15 pathway could further lower the activation threshold of NK cells and CD8^+^ T cells, and thereby facilitate recognition and elimination of virally infected and antigen-exposed tumor cells that resist direct oncolysis. Because we did not directly assess NK-cell activation markers or in vivo NK-cell dependence, these mechanistic links should be regarded as hypothesis-generating and supported by the prior IL-15/IL-15Rα literature, rather than as definitive proof obtained in the present study [39,40].

In line with this model, our in vivo data, systemic cytokine profiling, and FluoroSpot analysis suggest enhanced systemic immune activation, while tumor CD4/CD8 staining is suggestive of increased T-cell presence within the TME, accompanied by changes in circulating IL-2 and IFN-γ-producing cells that are compatible with effector lymphocyte extravasation and tumor-directed trafficking [31,41]. All groups share early suppression of IFN-γ, IL-1α, and IL-3, reflecting vaccinia-encoded immunoevasins, including the B8R IFN-γ decoy receptor and vCKBP chemokine-binding proteins [42]. The combination uniquely adds early co-elevation of IL-5 and IL-11—absent from VV-mIL15Rα alone (which suppresses IL-11) and inverted in VV-HSVtk alone (which suppresses IL-6). This divergence suggests that the addition of GCV-driven immunological cell death may contribute an extra layer of inflammatory signaling beyond that induced by the virus alone. The selective suppression of IL-9 only in combination groups is compatible with a more profound remodeling of certain regulatory and stromal compartments requiring simultaneous activity of both viral transgenes [43].

By the fifth injection, the shared rise in IL-17A and fall in TSLP across all effective groups was consistent with reduced stromal-driven immune tolerance. TSLP, produced by tumor-associated stroma, drives tolerogenic dendritic cell programming that suppresses effector T cell differentiation; its cumulative suppression by repeated virotherapy may facilitate Th17 expansion and antitumor neutrophil and γδ T cell activity. Likewise, concurrent downregulation of IL-11—a CAF-derived STAT3 activator that promotes tumor survival and pre-metastatic niche formation [44,45]—is in line with attenuation of CAF-driven stromal support in the 4T1 model. Paradoxically, late IFN-β suppression in HSVtk-containing groups could reflect a successful shift from antiviral to a more tumor-directed immune mode, facilitated by vaccinia E3L and K3L gene products that antagonize PKR-mediated IFN-β signaling [46] and allow sustained intratumoral viral replication [44,47,48].

The late-phase IL-5 elevation, co-occurring with IL-17A rise and TSLP suppression, may indicate conditions favoring eosinophil mobilization as a non-classical cytotoxic arm. Tumor-infiltrating eosinophils have been reported to kill tumor cells via perforin, granzyme, and reactive oxygen species, and remodel the TME through CXCL9/CXCL10 secretion that amplifies CD8^+^ T cell recruitment [49,50]. IL-22 elevation in HSVtk-containing groups is likewise compatible with increased sensitivity of residual tumor cells to immune killing via upregulating MHC class I and NK cell stress ligands. In the absence of direct eosinophil or neutrophil quantification in our study (semi-quantitative analysis of eosinophils represented in Supplementary Figures S13 and S14), we interpret these cytokine changes as suggestive, but not conclusive, for coordinated Th2/Th17-associated effector engagement. Together, these signals suggest the emergence of a multi-arm effector state involving cytotoxic T cells, NK cells, and, potentially, myeloid and granulocytic populations—a pattern that aligns with the pronounced tumor volume regression and durable control observed in a subset of animals, although specific cellular contributions remain to be dissected in future work [51,52]. Notably, oncolytic virotherapy has been shown in other settings to reprogram the TME in ways that support CD4^+^ T-cell memory formation, for example, via an IL-6Rα–Bcl6 axis in glioblastoma models, further supporting the concept that local oncolytic remodeling can imprint long-term systemic immunity. Similarly, oncolytic herpes simplex virus virotherapy has been reported to substantially modulate the intratumoral immune landscape, highlighting TME remodeling as a shared feature across distinct oncolytic platforms [53,54,55].

These systemic changes align with the local histological and immunohistochemical findings. The triple combination with GCV produced the most extensive tumor necrosis and the highest density of intratumoral CD4^+^ and CD8^+^ T cells, whereas tumors from control animals were essentially devoid of T-cell infiltrates. The HSVtk/GCV group also displayed CD4^+^ and CD8^+^ cells within the tumor, but at lower abundance than in the triple combination group, indicating that metabolic debulking alone can partially remodel the TME, whereas more pronounced conversion of “cold” 4T1 tumors into a T-cell-inflamed state appears to require concomitant IL-15 pathway targeting. This pattern is consistent with earlier studies of IL-15/IL-15Rα-armed OVs, where increased CD8^+^ T-cell infiltration correlated with improved tumor regression and survival [11]. The more heterogeneous distribution of CD4^+^ and CD8^+^ cells observed in the single-agent VV-HSVtk and VV-mIL15Rα groups suggests spatially variable immune engagement, mirroring the mixed cytokine profiles and underscoring the importance of combining metabolic and immunologic mechanisms within a single therapeutic scheme.

Histological examination of the livers and spleens from treated animals revealed no overt pathological abnormalities following repeated intratumoral administration, confirming that the recombinant LIVP variants did not produce detectable off-target organ toxicity under the conditions tested [11]. This safety profile is consistent with the established tropism of attenuated vaccinia virus strains for metabolically active tumor tissue and further supports the translational potential of this platform.

When viewed in the context of related approaches, our data support a model in which integrating HSVtk/GCV suicide gene therapy with IL-15Rα-targeted immunomodulation within a vaccinia backbone yields additive or synergistic benefits over either strategy alone. IL-15- and IL-15/IL-15Rα-armed OVs based on vaccinia, vesicular stomatitis virus, myxoma virus, and adenoviral vectors have demonstrated the ability to expand cytotoxic lymphocytes and generate systemic tumor immunity, particularly in combination with PD-1/PD-L1 or CTLA-4 blockade [6,56,57,58,59]. In parallel, HSVtk/GCV systems delivered by adenoviral or retroviral vectors have shown potent local tumor killing and bystander effects in multiple preclinical models and early clinical studies [20]. Our work brings these principles together in a single LIVP-based platform and demonstrates efficacy in a stringent 4T1 triple-negative breast cancer model that is resistant to many immunotherapeutic interventions. Notably, we used two coordinated but genetically distinct viral strains, separating metabolic and cytokine-receptor functions. This division-of-labor strategy, also explored in previous work [11], offers modularity and may reduce fitness costs associated with large multigenic inserts. In conclusion, active cytolytic activity—accompanied by bystander killing and immunogenic cell death—represents a critical complement to immunotherapy with an oncolytic vaccinia virus biovariant expressing an immunomodulatory transgene, as monotherapy likely fails to destroy a sufficient mass of malignant cells to prime a systemic, cell-mediated antitumor immune response.

## 4. Materials and Methods

### 4.1. Generation of Recombinant Viruses

LIVP strain with disruption of TK and expression of tagRFP reporter gene under the control of 7.5k promoter (VV-RFP) and mIL-15Rα were constructed and described previously [2,41]. To construct an HSVtk-expressing variant, DNA was extracted from HEK293T cells infected with HSV type 1 at MOI = 1, 24 h after infection, using the phenol-chloroform method as previously described [49]. Next, using Q5 High Fidelity polymerase (NEB, Ipswich, MA, USA), the HSVtk gene was amplified and cloned by sticky-end ligation into the recombination plasmid previously developed at the Cell Proliferation laboratory EIMB RAS [2]. Transfection for recombination was performed using PEI in HEK293T cells as described before [60]; the cells were infected with the LIVP strain at MOI = 1. TK-disrupted recombinant variants were selected on Rat-2 TK-minus cells (deficient in the TK expression) treated with 2-bromodeoxyuridine after infection at an MOI of 1 of the virus variants gained after transfection, separated by plaque assay, and cloned. Insertion was confirmed by sequencing of the amplified TK locus from viral genomic DNA. Recombinant viruses were propagated in BHK-21 cells and purified by the sucrose gradient method for in vitro and in vivo experiments [2].

### 4.2. Cell Lines and Culture Conditions

Baby hamster kidney BHK-21 (CCL-10 ATCC) cells, 4T1 murine breast adenocarcinoma cells (CRL-3406 ATCC), and a panel of human cancer cell lines—including HeLa, HEK293T, HCT116, NCI-H460, TOV21G, DU145, MCF7, and C33A—were obtained from the collection of the laboratory of cell proliferation (EIMB RAS, Moscow, Russia). CT2A murine glioma cells were kindly provided by Dr. Aleksei A. Stepanenko (Department of Fundamental and Applied Neurobiology, Serbsky National Medical Research Center for Psychiatry and Narcology, Moscow, Russia). Cells were maintained in Dulbecco’s Modified Eagle’s Medium (DMEM/Glutamax; Servicebio, Wuhan, China), supplemented with 10% fetal bovine serum (FBS, HyClone, Cytiva, Logan, UT, USA) and penicillin–streptomycin at standard concentrations (PanEco, Moscow, Russia). Human NK cells for co-culture assays were isolated from healthy donor PBMCs and kindly gifted by Professor Vladimir Baklaushev.

### 4.3. In Vitro Cytotoxicity and NK Cell Co-Culture

Cell viability was evaluated using the Resazurin (Alamar Blue) assay. Cells were seeded in 96-well plates and infected with VV-HSVtk-RFP or VV-RFP at a wide range of MOI (0.001, 0.01, 0.1, 1, 10, and 100). At 24 h post-infection, GCV was added at concentrations of 12.5, 25, 50, and 100 μM. Resazurin (0.15 mg/mL; Sigma-Aldrich, Burlington, Massachusetts, U.S.) was added to each well, and absorbance was measured at 24, 48, 72, and 96 h. For NK cells, co-cultivation MOI = 1 was used; pre-infected cells were added with Effector:Target (E:T) cells at a ratio of 5:1 at 24 h post-infection alongside GCV treatment.

### 4.4. RT-qPCR

Total RNA was isolated from 4 × 10^5^ cells infected with VV-HSVtk using ExtractRNA reagent and CleanRNA Standard kit (Evrogen, Moscow, Russia), followed by DNase I treatment (Thermo Fisher Scientific, Waltham, MA, USA). cDNA was synthesized using SuperScript III Reverse Transcriptase (Invitrogen, Carlsbad, CA, USA) and random octamer primers per the manufacturer’s protocol. qPCR was conducted on a CFX96 Touch System (Bio-Rad, Hercules, CA, USA) with qPCRmix-HS SYBR (Evrogen, Moscow, Russia) and gene-specific primers (Table S1), validated for specificity via melting curve analysis (single peaks, no non-specific products) and efficiency through serial cDNA dilutions (R^2^ > 0.98 for all pairs). Relative expression was calculated by the comparative ΔCt method [49], using β-actin as an internal control. Fold changes in gene expression were calculated using the 2-ΔCt method.

### 4.5. Assessment of Therapeutic Efficacy In Vivo

The antitumor efficacy was evaluated in the syngeneic BALB/c murine 4T1 mammary carcinoma model. Animals were divided into eight essential treatment groups (Table 1).

All animal procedures were approved by the EIMB Institutional Animal Care and Use Committee (Protocol No. 1 issued on 5 March 2025). Female and male 6-week-old Balb/c mice were used for 4T1 models. Once tumors reached approximately 50 mm^3^, mice were randomized into eight groups: Saline, GCV-only, VV-HSVtk, VV-mIL15Rα, VV-HSVtk/GCV, VV-mIL15Rα/GCV, VV-HSVtk + VV-mIL15Rα, and triple combination (VV-HSVtk + VV-mIL15Rα + GCV). Viruses were administered intratumorally five times every 4 days (2 × 10^7^ PFU of total dose). In GCV-treated groups, GCV (50 mg/kg) was injected intraperitoneally 48 h after each virus administration. Tumor volume was measured by calipers every other day and calculated as V = (Length × Width^2^)/2.

### 4.6. PBMC Isolation and Preparation

Mouse peripheral blood was harvested into sterile microcentrifuge tubes pre-treated with EDTA as an anticoagulant, and PBMCs were isolated via Ficoll–Paque density gradient centrifugation (350× *g*, 20 min, no brake), followed by washing in PBS + 2% FBS. Cells were resuspended at 5–10 × 10^6^/mL in freezing medium (90% FBS + 10% DMSO), aliquoted into cryovials, and cryopreserved using a controlled-rate freezer (−1 °C/min to −80 °C) prior to transfer to liquid nitrogen for retrospective ELISPOT analysis. On the day of the experiment, they were gently thawed. The resulting PBMCs were washed and resuspended in complete DMEM medium supplemented with 10% fetal bovine serum (FBS) and 1% penicillin–streptomycin at standard concentrations. Cell viability and density were determined via trypan blue exclusion using an automated cell counter Luna II (RWD Life Science, Shenzhen, China).

### 4.7. Fluorospot Analysis

To quantify the frequency of polyfunctional T-cell populations, a multiplex FluoroSpot immunoassay (Mabtech AB, Stockholm, Sweden) was performed to detect IFN-γ, IL-2, and TNF-α activated cells simultaneously at the single-cell level. The plates were washed and blocked with complete DMEM medium containing 10% FBS for at least 30 min at room temperature. Defrizzed PBMCs were seeded at a density of 2.5 × 10^5^ cells/well in triplicate. To support the detection of cytokines from pre-activated T cells, monoclonal anti-CD28 (0.1 μg/mL) was added to all cell-containing wells as a costimulatory signal. Wells containing recombinant mouse IFN-γ, IL-2, and TNFα were processed in parallel as positive controls to validate the performance of the detection reagents and the accuracy of the fluorescent signal co-localization. After 24 h of incubation at 37 °C in 5% CO_2_, the cells were removed, and the plates were treated with a cocktail of specific detection antibodies: biotinylated anti-IFN-γ (R4-6A2), BAM-conjugated anti-IL-2 (MT156B6), and WAV-conjugated anti-TNF-α (MT25C5). After 2 h, the membranes were washed and incubated for 1 h with a fluorophore-conjugated secondary antibody cocktail consisting of Streptavidin-490, anti-BAM-550, and anti-WAV-640. Finally, the plates were treated with a fluorescence enhancer for 15 min, dried in the dark, and analyzed using an automated reader. Spot-forming cells (SFCs) and polyfunctional “multi-spot” populations were enumerated by identifying spatially co-localized signals across the 490 nm, 550 nm, and 640 nm channels, with values expressed as SFCs per 3 × 10^5^ PBMCs after background subtraction.

### 4.8. Serum Collection and Processing

To monitor the systemic cytokine response throughout the treatment course, blood samples were collected from the tail vein of 4T1-tumor-bearing mice 24 h after each of the five viral administrations. To ensure adequate blood flow and minimize animal distress, mice were briefly warmed under a heat lamp or in a warming chamber prior to the procedure. Approximately 50–100 μL of whole blood was collected into sterile microcentrifuge tubes pre-treated with EDTA as an anticoagulant. The samples were then subjected to centrifugation at 2000× *g* for 10 min at 4 °C to separate the cellular components. The resulting supernatant was carefully aspirated, and the serum fractions were aliquoted and stored at −80 °C to preserve cytokine stability until further multiplex analysis. All procedures were performed in accordance with institutional animal welfare guidelines to ensure minimal distress to the mice during repeated sampling.

### 4.9. Multiplex Cytokine Quantification (LEGENDplex™)

Systemic cytokine concentrations were quantified using bead-based multiplex immunoassays. Serum samples were analyzed using the LEGENDplex™ Mouse Cytokine Panel 2 (Standard V02) and the LEGENDplex™ Mouse Th Cytokine Panel (12-plex) with V-bottom plate (VbP) (Cat. No. 741038, Version V03, 100 tests) (BioLegend, San Diego, CA, USA) according to the manufacturer’s instructions.

The combined panels allowed for the simultaneous detection of a broad range of analytes, including pro-inflammatory cytokines, Th-associated mediators, and immunoregulatory factors. Briefly, serum samples were incubated with cytokine-specific capture beads, which are distinguished by their size and internal fluorescence intensities. Following the primary incubation, biotinylated detection antibodies were added to form sandwich complexes, followed by the addition of streptavidin–phycoerythrin (SA-PE) to provide a fluorescent signal proportional to the amount of bound analyte.

Data acquisition was performed using a BD LSRFortessa™ flow cytometer (Waters Bioscience, Milford, MA, USA). A minimum of 2000 beads per analyte was collected to ensure statistical robustness. The raw fluorescence intensities were processed using the LEGENDplex™ Data Analysis Software, where cytokine concentrations were calculated by interpolation from a five-parameter logistic (5PL) standard curve. All samples were run in duplicate, and values were expressed in pg/mL.

### 4.10. IHC Analyses

For histological analysis, subcutaneous tumors, livers, and spleens were collected and fixed in 4% paraformaldehyde at 4 °C overnight. In 16 h, tissue samples were embedded in paraffin, and serial 5 μm sections were prepared using a rotary microtome (Leica Microsystems, Wetzlar, Germany), and stained with hematoxylin and eosin (H&E) according to standard protocols [48].

Tumor immune cell infiltration was assessed on coronal 50 μm cryosections (Leica VT1200 S vibratome), permeabilized in 0.1% Triton X-100, and blocked with 1% goat serum. Sections were incubated overnight at 4 °C with Alexa Fluor 488 anti-mouse CD4 (100423, Biolegend) and eFluor 660 CD8a (AMC908, eBioscience, San Diego, CA, USA). Images were acquired using a Nikon A1R confocal laser scanning microscope (Nikon, Tokyo, Japan).

### 4.11. In Vivo Synergy Analysis

Synergy for in vivo tumor volume data was evaluated using the invivoSyn framework [61]. Longitudinal tumor volume measurements from each treatment group (VV-HSVtk/GCV, VV-mIL15Rα, and VV-HSVtk/VV-mIL15Rα/GCV) were imported into invivoSyn, and synergy was assessed using the Bliss independence reference model applied to tumor growth inhibition over time. For each time course, invivoSyn estimated the Bliss-expected additive tumor growth trajectory based on the monotherapy groups and compared it with the observed trajectory under combination treatment to derive a synergy *p*-value (psyn). A psyn below 0.05 was considered evidence of a statistically significant more-than-additive (synergistic) interaction.

### 4.12. Statistical Analysis

Statistical processing was performed using GraphPad Prism version 10.1.2 (GraphPad Software, San Diego, CA, USA). Data are presented as mean ± SD. All experiments were performed in at least three independent biological replicates. For comparisons between multiple experimental groups, a one-way or two-way analysis of variance (ANOVA) was employed, followed by appropriate post hoc tests for multiple comparisons. The log-rank test was used for survival analysis. Differences were considered statistically significant at: *, *p* < 0.05; **, *p* < 0.01; ***, *p* < 0.001; and ****, *p* < 0.0001.

## 5. Conclusions

Collectively, these findings establish that the coordinated deployment of HSVtk/GCV-mediated cytotoxic bystander activity and mIL-15Rα-driven immune co-stimulation within a recombinant LIVP vaccinia virus platform produces complementary and mutually reinforcing antitumor mechanisms. Metabolic debulking generates the immunogenic cell death and antigen landscape required for adaptive immune priming, while IL-15 trans-presentation sustains the T cell effector populations needed to execute and perpetuate that response. The result is delayed tumor progression/therapeutic regression and prolonged survival in the aggressive syngeneic 4T1 model—an outcome that neither component achieved as monotherapy. Beyond their direct therapeutic significance, these results provide a mechanistic rationale for the broader development of multimodal armed vaccinia virus platforms in which direct cytotoxicity and immunostimulatory transgene expression are engineered to act in concert. Future studies should evaluate this combination in orthotopic and metastatic settings, explore the durability of the immunological memory generated, and assess synergy with systemic checkpoint inhibition—an approach whose efficacy is likely to be substantially enhanced by the TME remodeling demonstrated here.

## Figures and Tables

**Table 1:** Treatment groups.

Group	Treatment Components	Primary Function
1	Control (saline)	Baseline
2	Ganciclovir (GCV)	Prodrug toxicity control
3	VV-HSVtk	TAA release
4	VV-mIL15Rα	Immune stimulation
5	VV-HSVtk/GCV	GDEPT + Oncolysis
6	VV-mIL15Rα/GCV	Immune stimulation + (GCV control)
7	VV-HSVtk + VV-mIL15Rα	Combined oncolysis/TAA/immune
8	VV-HSVtk + VV-mIL15Rα/GCV	GDEPT + Synergistic immune boost

## Data Availability

The original contributions presented in this study are included in the article/Supplementary Material. Further inquiries can be directed to the corresponding authors.

## Supplementary Materials

The supporting information can be downloaded at: https://www.mdpi.com/article/10.3390/ijms27135838/s1.

## Abbreviations

The following abbreviations are used in this manuscript:

GCV	Ganciclovir
DAMPs	Damage-associated molecular patterns
HSVtk	Herpes simplex virus thymidine kinase
i.t.	Intratumoral
i.p.	Intraperitoneal
IHC	Immunohistochemistry
LIVP	Lister (strain) Institute of Viral Preparation
MHC	Major histocompatibility complex
MOI	Multiplicity of infection
NK	Natural killer
OVs	Oncolytic viruses
TAAs	Tumor-associated antigens
TCID50	50% tissue culture infectious dose
TK	Thymidine kinase
TME	Tumor microenvironment
TNBC	Triple-negative breast cancer
VV	Vaccinia virus
