# Supplementary material for: Synergistic Oncolytic Effect of HSVtk- and IL-15Rα-Armed Vaccinia Viruses Inducing Systemic Antitumor Immunity
Source: Int J Mol Sci. 2026 Jun 28;27(13):5838. doi: 10.3390/ijms27135838 (PMC13361383; doi:10.3390/ijms27135838)
Supplement: Supplementary file 1 [file ijms-27-05838-s001.zip › ijms-4338433-supplementary.pdf]

International Journal of
Molecular Sciences

MDPI

Supplementary Materials

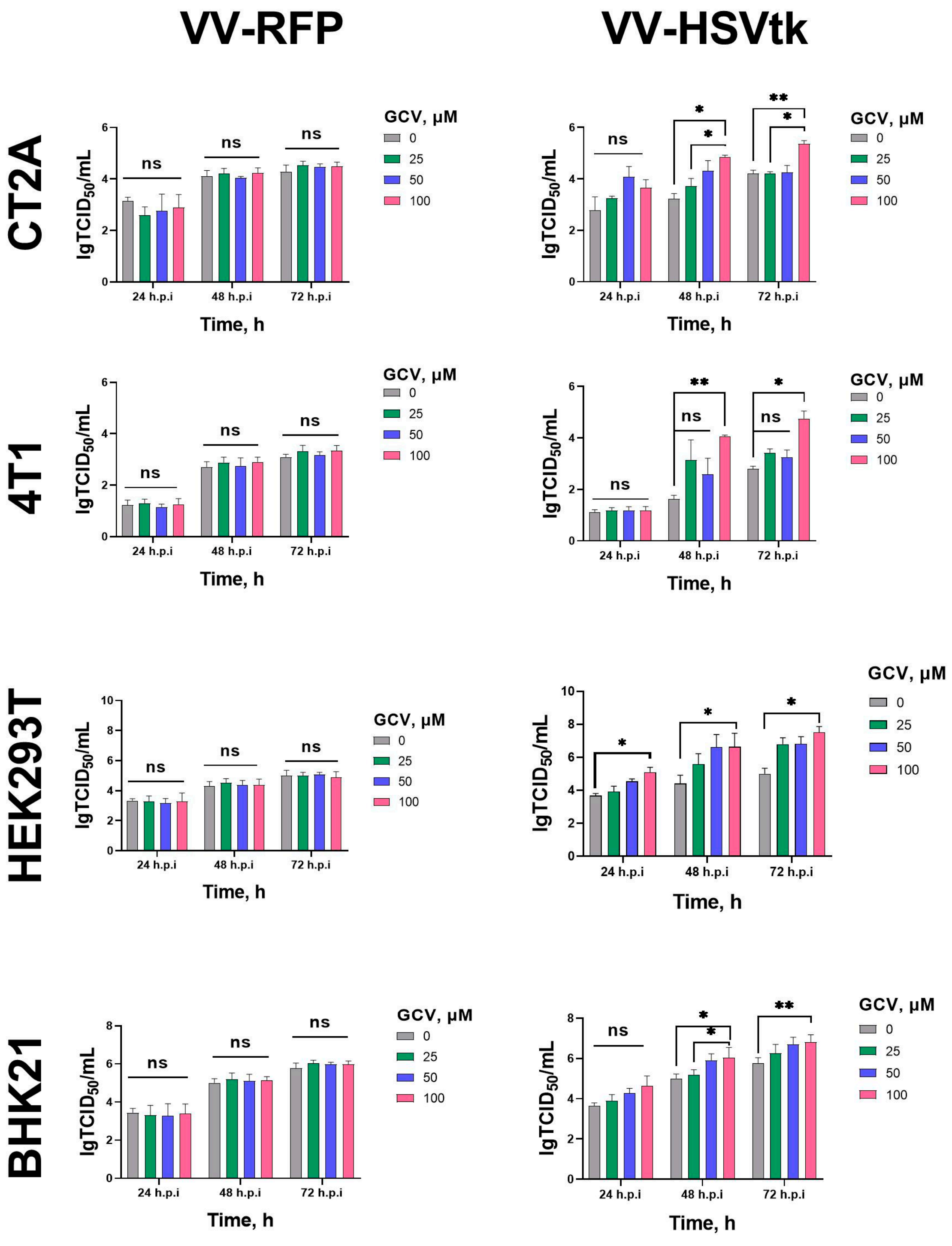

Figure S1. Sensitivity of cells to VV-HSVtk and VV-RFP in presence or absence of GCV. Statistical analysis was performed using two-way ANOVA; * p<0.05, ** p< 0.01.

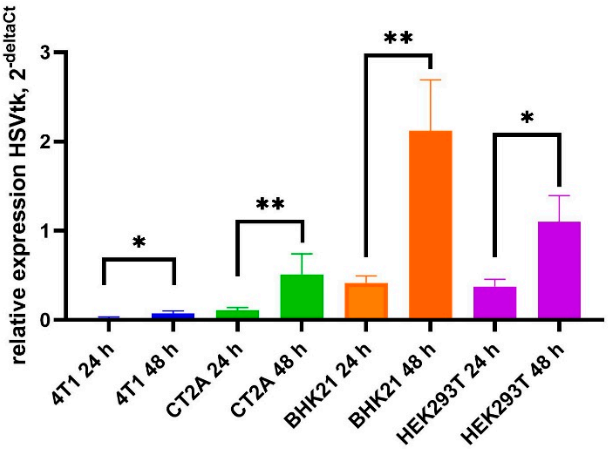

Figure S2. Relative expression of VV-HSVtk in infected cells 24 and 48 hours post infection. For statistical analysis, the Mann–Whitney test was used; *p < 0.05, **p < 0.01.

Table S1. Primers for RT-PCR

#	**Primer name**	**Sequence**
1	Human ACTB for	CAG CAG ATG TGG ATC AGC AAG
2	Human ACTB rev	GCA TTT GCG GTG GAC GAT
3	Mouse ACTB for	CTC CTG AGC GCA AGT ACT CTG TG
4	Mouse ACTB rev	TAA AAC GCA GCT CAG TAA CAG TCC
5	Hamster ACTB for	GTG CTA TGT TGC CCT GGA CT
6	Hamster ACTB rev	GCT CGT TGC CAA TGG TGA TG
7	HSVtk for	GTC CCC GGC CGA TAT CTC AC
8	HSVtk rev	AAC CAC CAC CAC GCA ACT G

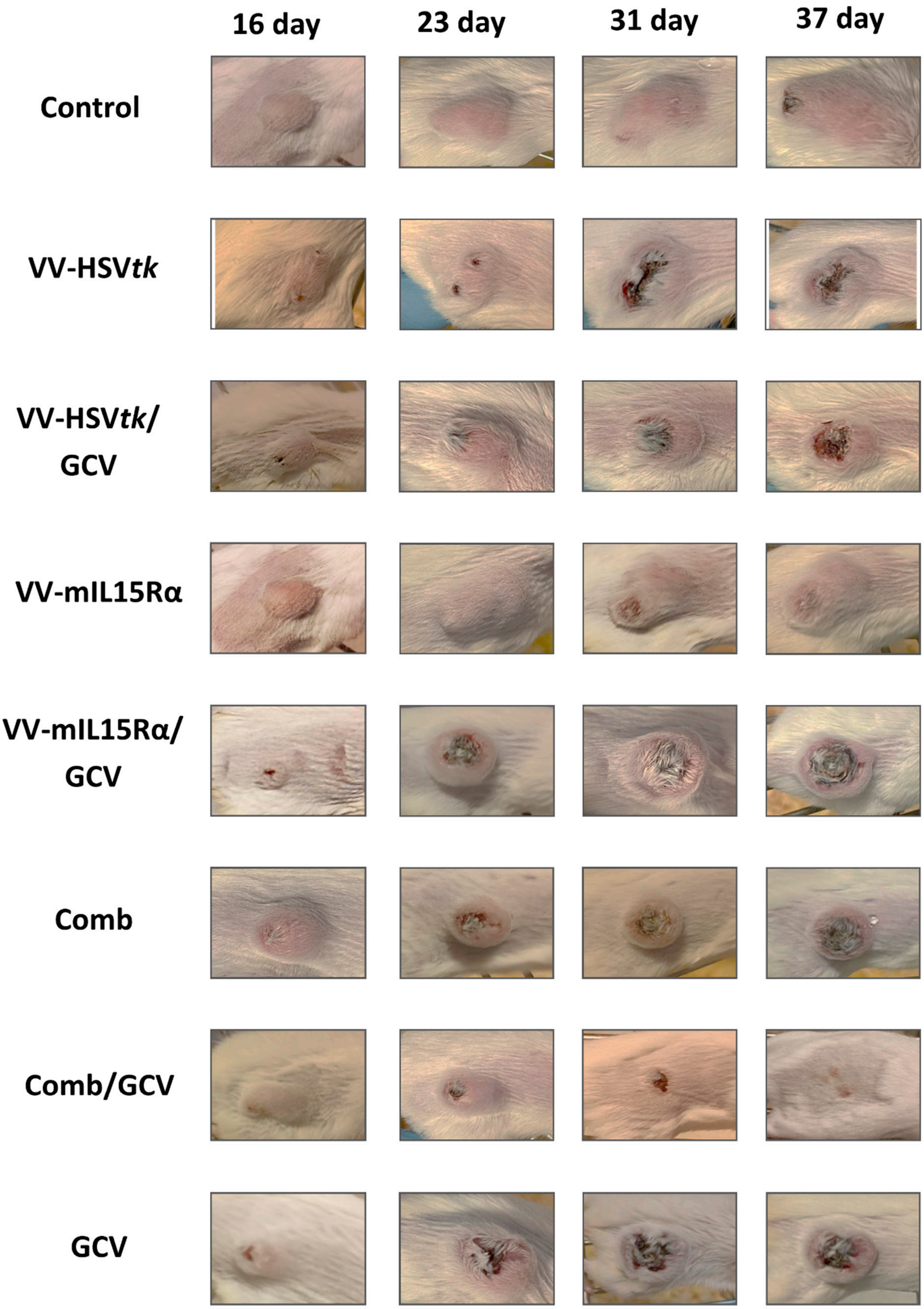

Figure S3. Macrophotographs of tumor regression after treatment at different time points.

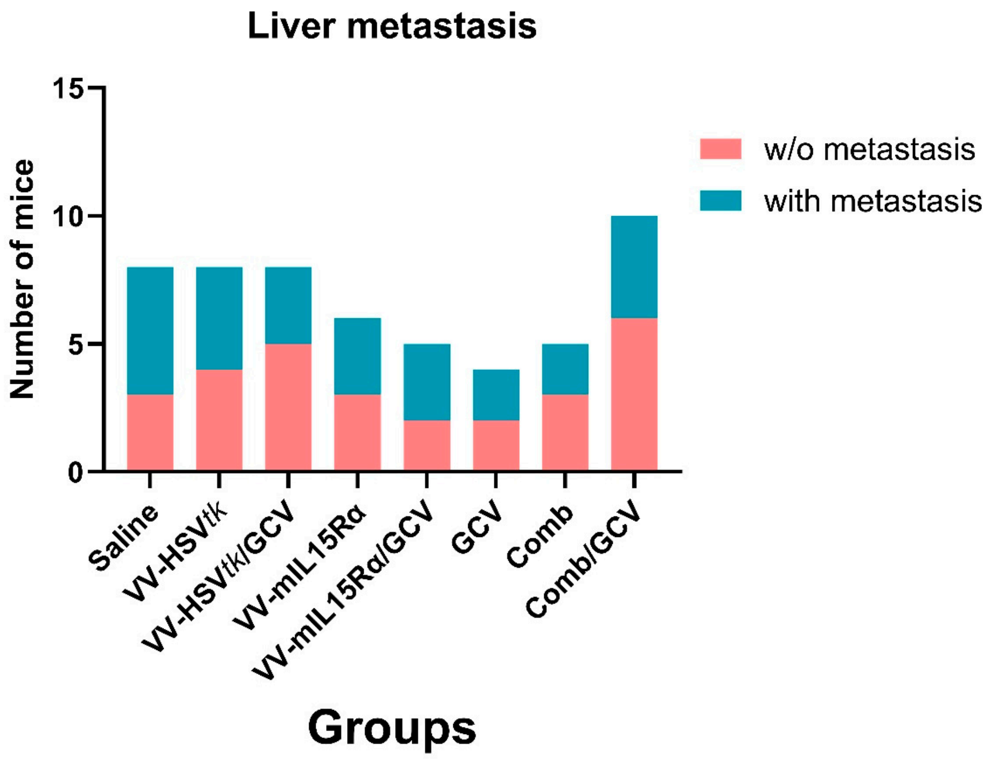

Figure S4. Liver metastasis (qualitative analysis) in treatment groups. Metastatic lesions in the liver were identified macroscopically on fixed organs and confirmed histologically after hematoxylin and eosin staining.

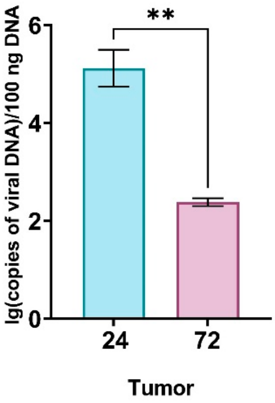

Figure S5. Viral genomic DNA presence in 4T1 tumors assessed 24 and 72 hours after injection. Tumors were collected from treated mice (n = 3 per time point) to determine viral presence and quantify genome DNA copy number by qPCR. For statistical analysis, the Mann–Whitney test was used; **p < 0.01.

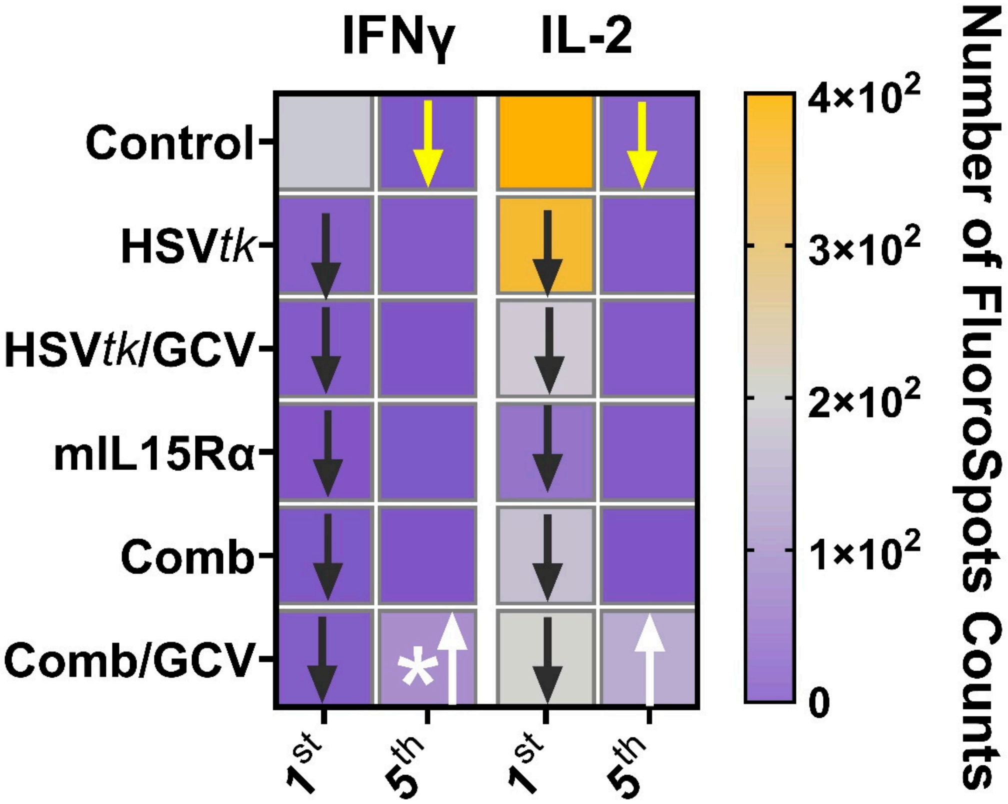

Figure S6. Assessment of cytokine dynamics in the PBMCs of treated animals measured by Fluorospot multiplex immunoassay. Numbers of spots are shown on the right side of the heatmap. The color intensity of each cell in the heatmap represents the mean cytokine positive cell count for the corresponding group and time point. Arrows denote changes greater than 30% that, due to the limited sample size, did not reach statistical significance but suggest biologically relevant trends. Yellow shading highlights changes observed in the control group between the first and fifth injections, whereas white arrows indicate increases and black arrows indicate decreases relative to the corresponding control group. Statistical analysis was performed using two-way ANOVA, *$p < 0.05$.

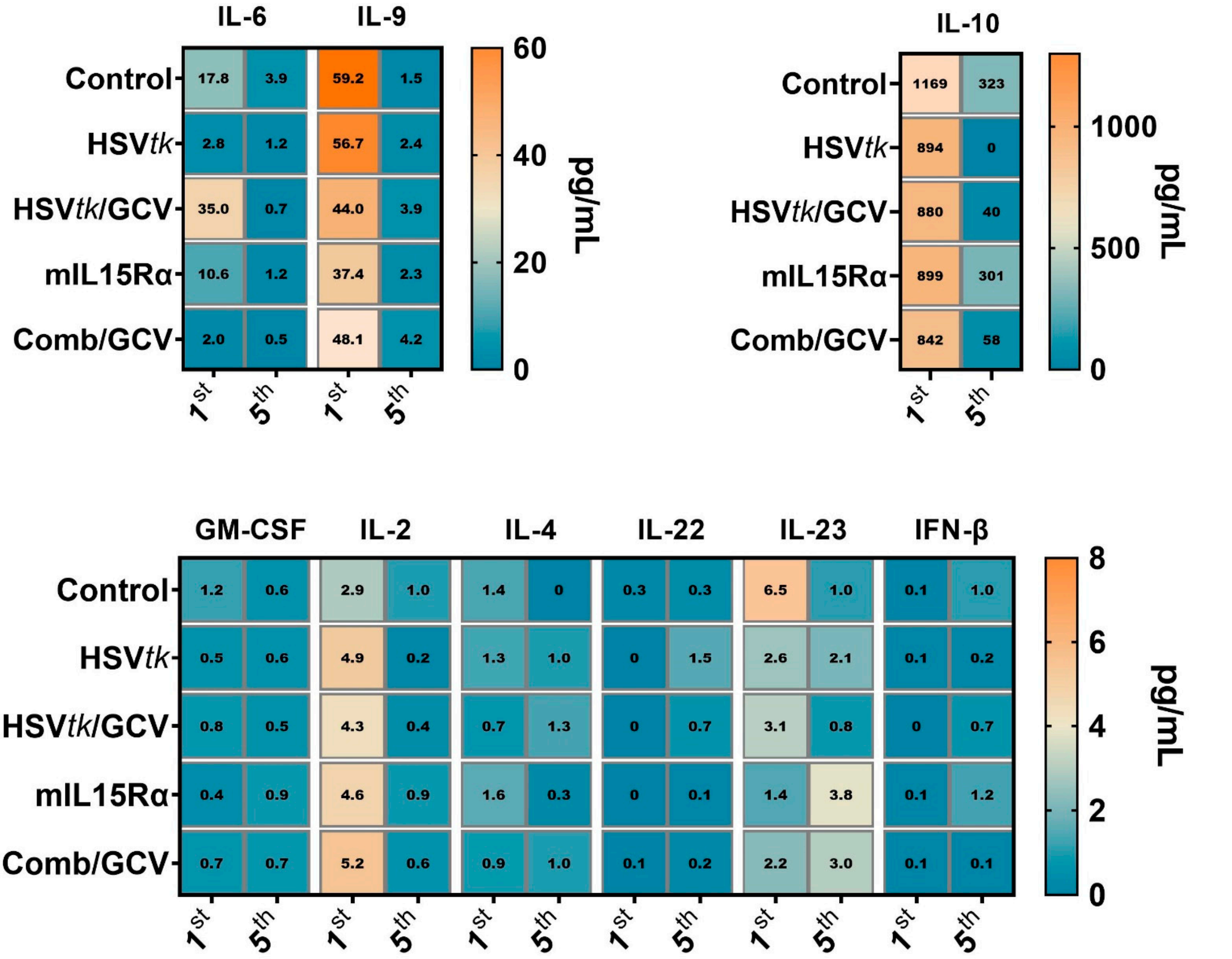

Figure S7. Assessment of cytokine dynamics in the serum of treated animals, grouped by average concentration from highest to lowest (left to right), with concentration scales shown on the right of the heatmap. The color intensity of each cell in the heatmaps represents the mean cytokine concentration for the corresponding group and time point.

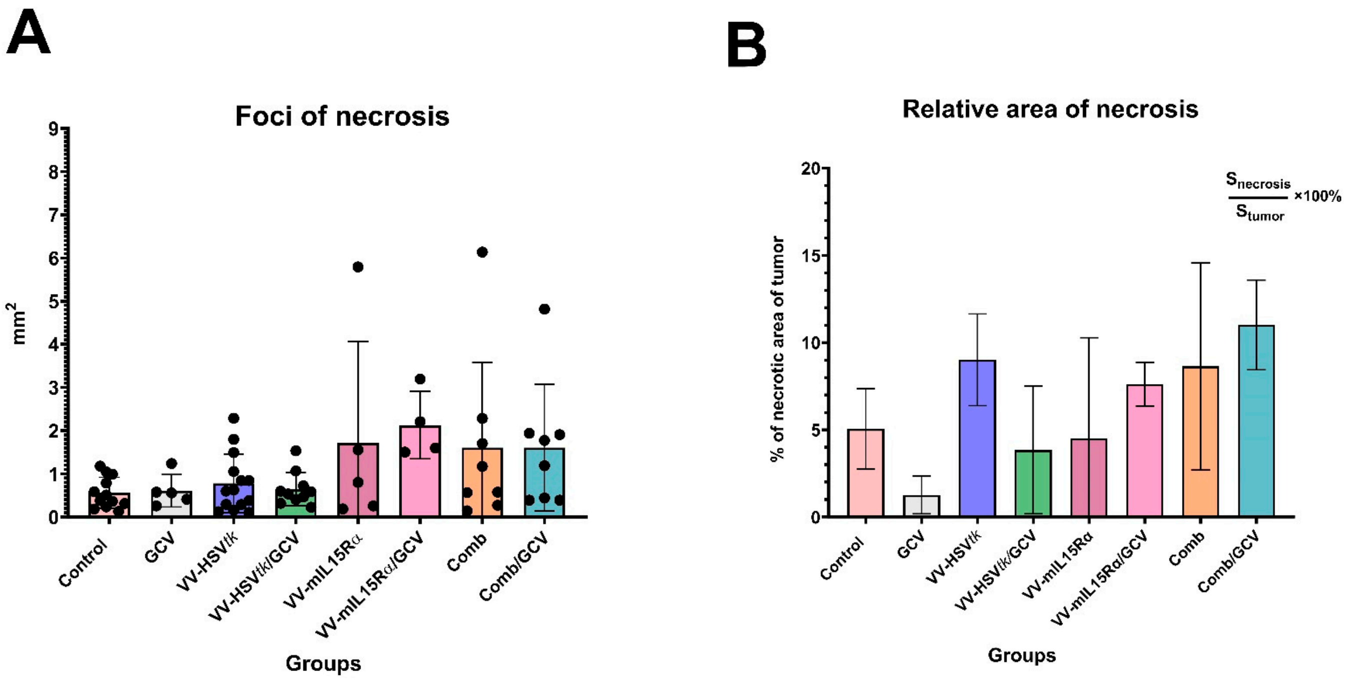

Figure S8. (a) Surfaces of 4T1 tumor necrosis foci in mm^2. Every dot represents a single area of necrosis. (b) Relative area of necrosis. Necrosis was assessed by histological analysis via hematoxylin–eosin–stained tumor sections at 35th day of experiment.

International Journal of
Molecular Sciences

MDPI

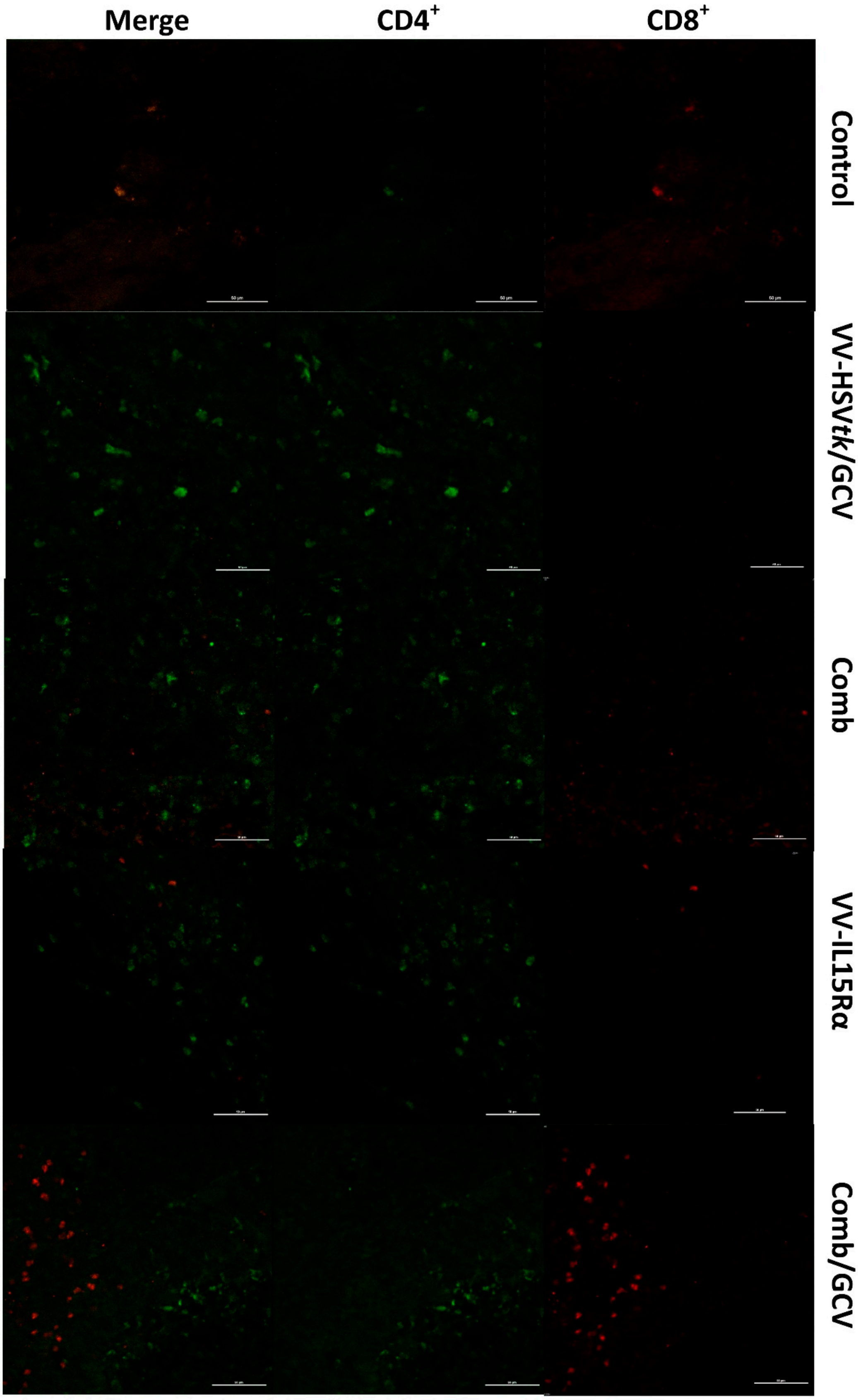

Figure S9. Immunofluorescent detection of $CD4^+$ and $CD8^+$ cells in 4T1 tumors after treatment. Representative fluorescence images show intensive infiltration of $CD8^+$ (red) and $CD4^+$ cells (green) in group with triple combination treatment. Scale bar, 50 μm.

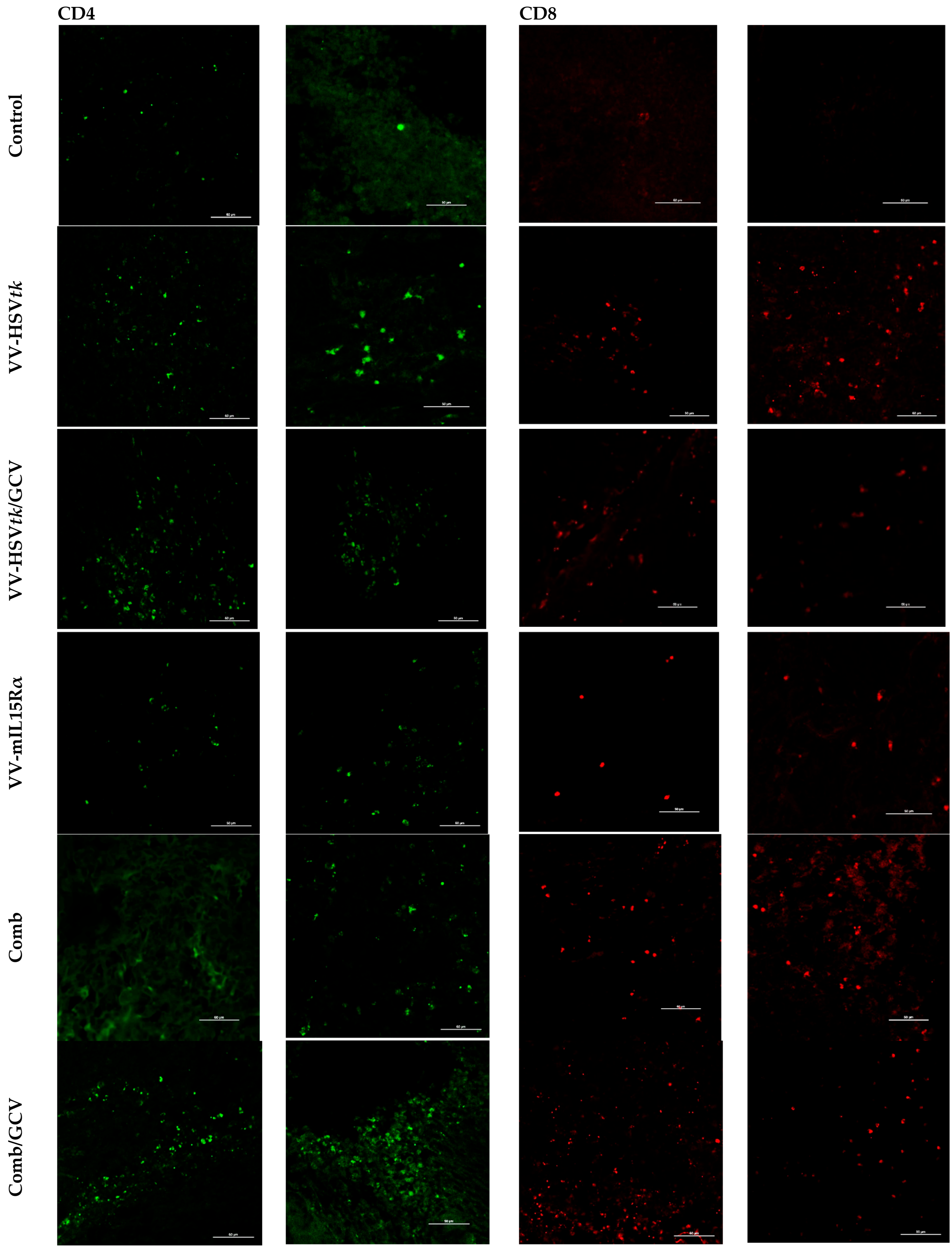

Figure S10. Immunofluorescent detection of CD4+ and CD8+ cells in 4T1 tumors after treatment (additional representative images). Representative fluorescence images show intensive infiltration of CD8+ (red) and CD4+ cells (green) in group with triple combination treatment. Scale bar, 50 μm.

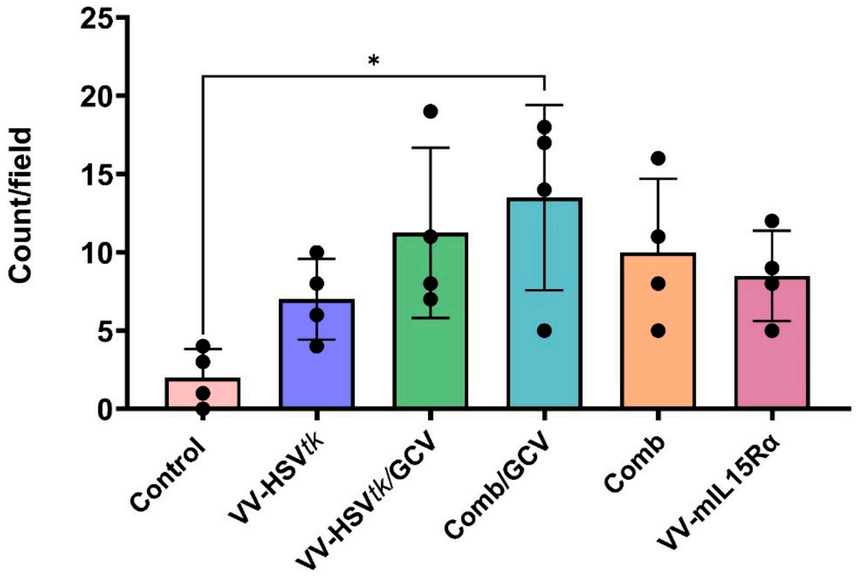

Figure S11. Tumor infiltrated CD4+ lymphocyte counts per field for each treatment group. Statistical analysis was performed using two-way ANOVA; *p < 0.05.

International Journal of Molecular Sciences

MDPI

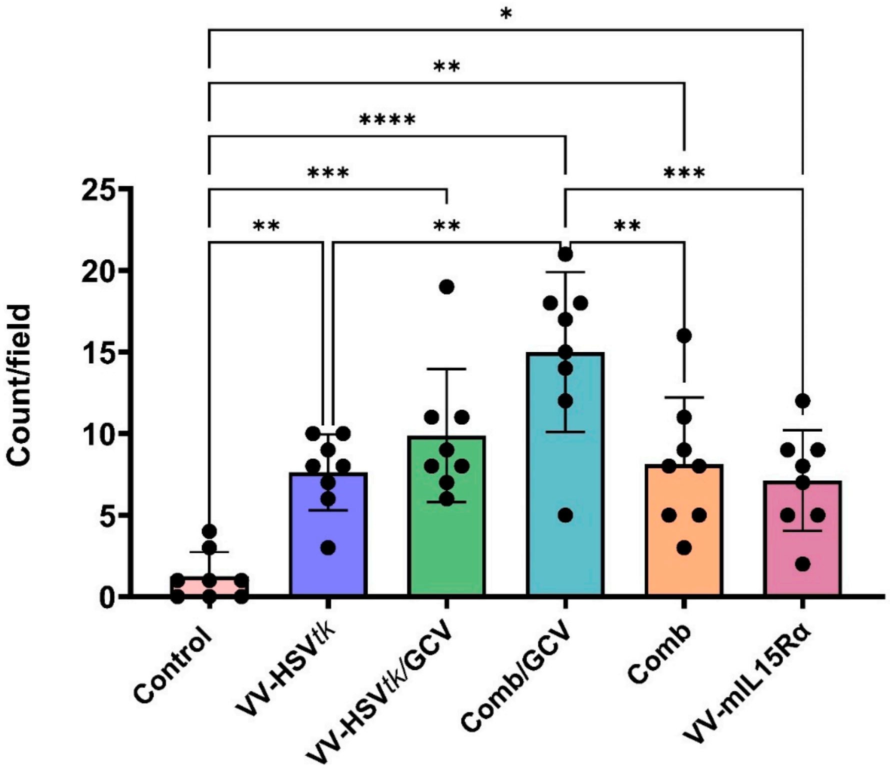

Figure S12. Tumor infiltrated $CD8^+$ lymphocyte counts per field for each treatment group. Statistical analysis was performed using two-way ANOVA; *p < 0.05, **p < 0.01, ***p<0.001, ****p<0.0001

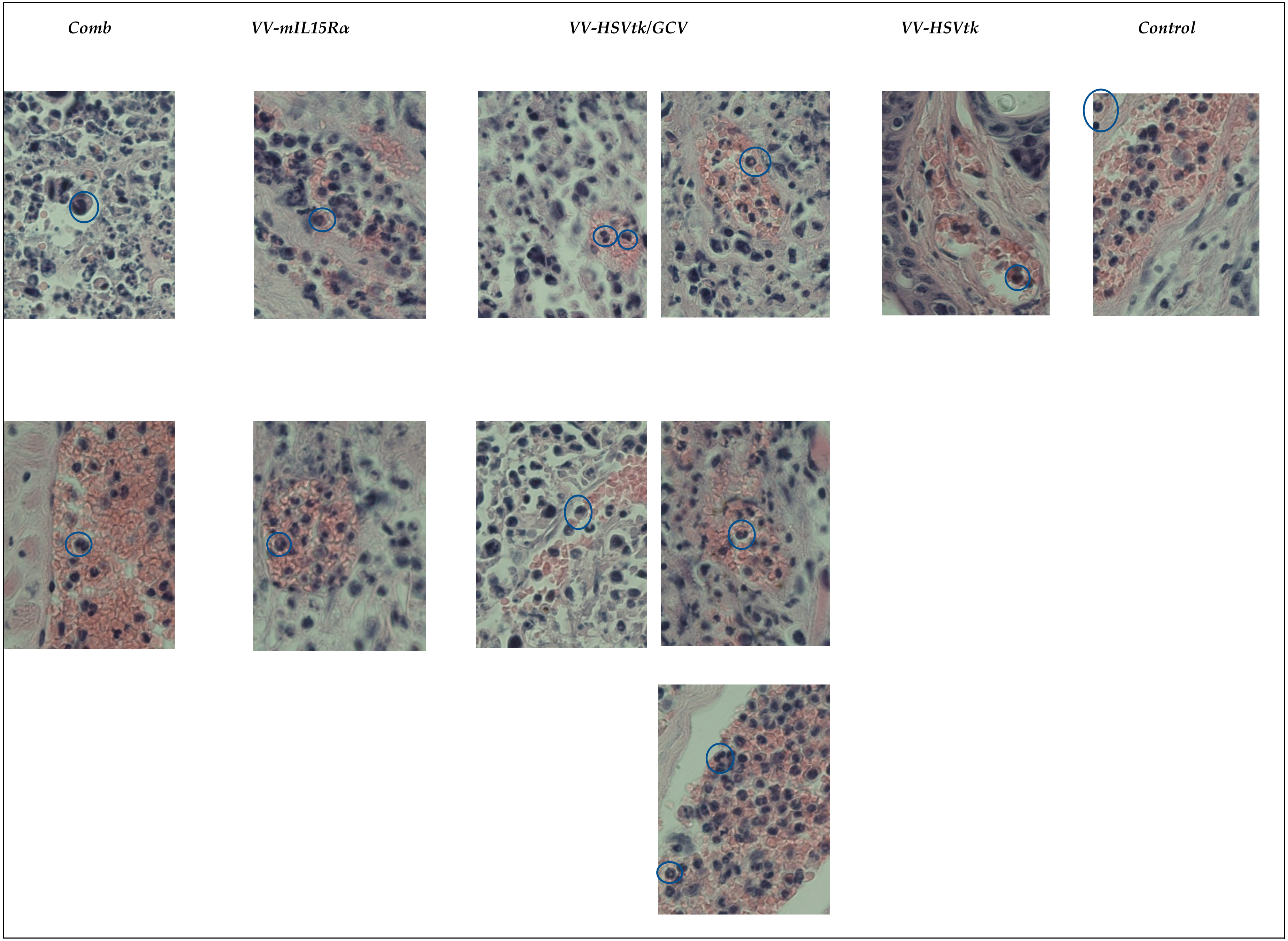
Comb
VV-mIL15Rα
VV-HSVtk/GCV
VV-HSVtk
Control

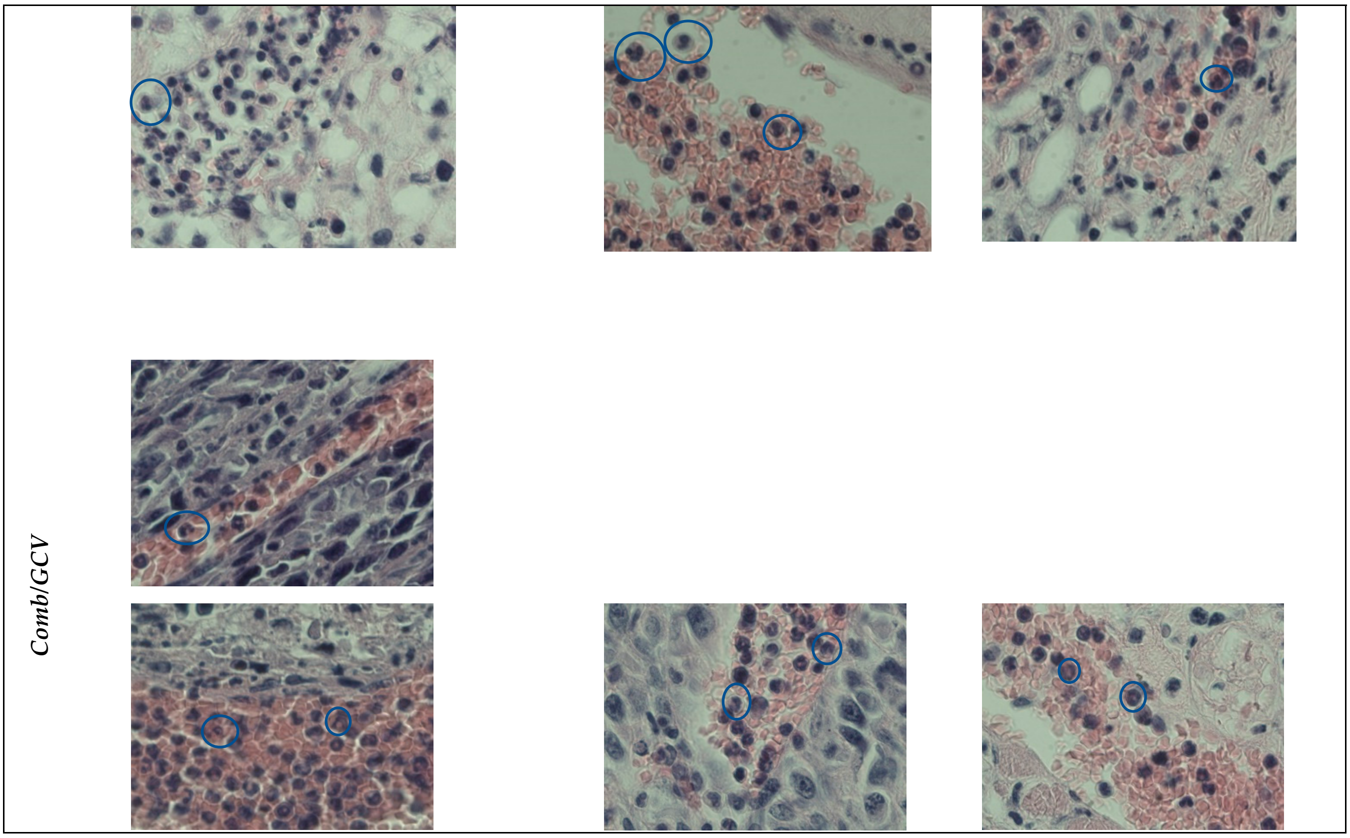

Figure S13. Panel of hematoxylin–eosin (H&E)-stained tumor microphotographs from panoramic whole-tumor scans as the only sections containing cells with morphological features characteristic of eosinophils. Samples were obtained from two mice per experimental group. Eosinophils are indicated by blue circles.

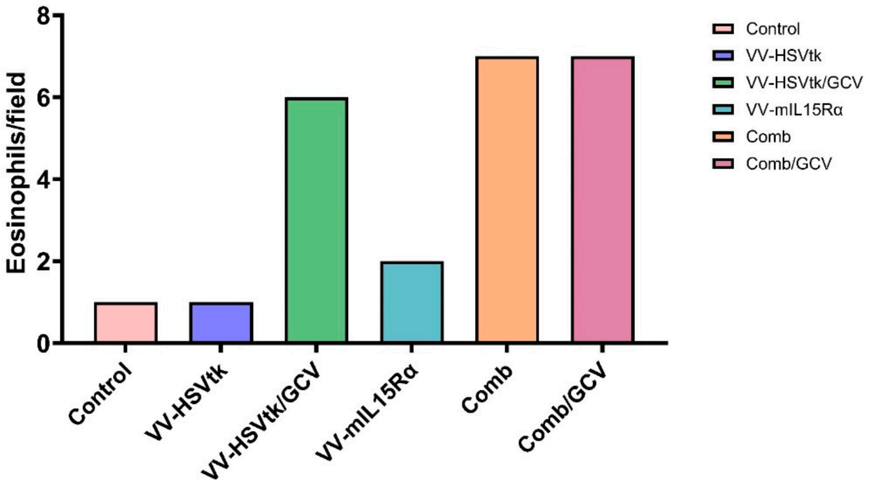

Figure S14. Quantification of eosinophils in panoramic whole-tumor section images from two mice per treatment group
